# Vaccine-induced ADCC-mediating antibodies target unique and overlapping envelope epitopes

**DOI:** 10.1186/1742-4690-9-S2-O39

**Published:** 2012-09-13

**Authors:** J Pollara, M Bonsignori, M Moody, M Alam, H Liao, K Hwang, J Pickeral, J Kappes, C Ochsenbauer, K Soderberg, TC Gurley, DM Kozink, DJ Marshall, JF Whitesides, D Montefiori, JE Robinson, J Kaewkungwal, S Nitayaphan, P Pitisuttithum, S Rerks-Ngarm, J Kim, N Michael, G Tomaras, BF Haynes, G Ferrari

**Affiliations:** 1 Duke University, Durham, NC, USA; 2 University of Alabama at Birmingham, Birmingham, AL, USA; 3 Tulane University School of Medicine, New Orleans, LA, USA; 4 Tropical Hygiene, Mahidol University, Bangkok, Thailand; 5 Armed Forces Research Institute of Medical Sciences, Bangkok, Thailand; 6 Clinical Tropical Medicine, Mahidol University, Bangkok, Thailand; 7 Department of Disease Control, Ministry of Public Health, Nonthaburi, Thailand; 8 US Military HIV Research Program, Rockville, MD, USA

## Background

Antibody Dependent Cellular Cytotoxicity (ADCC) may be a contributing factor of immune responses controlling HIV-1 replication. Understanding the epitopes recognized by ADCC-mediating antibodies is likely to be important for the development of an effective AIDS vaccine. We characterized the epitope specificity and breadth of the ADCC-mediating antibody response elicited by the RV144 vaccine regimen.

## Methods

Twenty-three monoclonal antibodies (mAbs) were isolated from 6 vaccine recipients either from IgG^+^ memory B cells cultured at near clonal dilution for 14 days (n=115,200) followed by sequential screenings of culture supernatants for HIV-1 gp120 Env binding, or from memory B cell (n=206,745) sorting for HIV-1 group M consensus gp140Con.S Env binding. Target cells infected with infectious molecular clones expressing Clade A/E (CM235), B (BaL), and C (DU422 and DU151) env were used to characterize the specificity and breadth of the 23 mAbs that display ADCC activity. We defined the epitope specificity of the isolated mAbs by mapping with B.MN and/or AE.92TH023 linear peptides in ELISA and with Fab-competition in ADCC assays.

## Results

Linear mapping revealed that 2 mAbs recognized the V2, and 1 mAb the V3 regions of the gp120. Nineteen (19) mAbs recognized conformational epitopes overlapping the C1 A32 epitope; one mAb (CH20) recognized a conformational epitope that was not blocked by any of the Fabs (A32, 19B, 17b) utilized in our assay. Fourteen of the 20 mAbs directed against conformational epitopes mediated ADCC against the clade B BaL Env; 4 recognized the clade C DU151 Env and 1 recognized the clade C DU422 Env.

## Conclusion

The RV144 vaccine regimen induced broadly-reactive ADCC Abs that recognized both unique and overlapping regions of gp120. The mAbs with the greatest breadth may be useful for passive protection trials in rhesus macaques. If protective in non-human primates, the epitopes recognized by these mAbs may inform immunogen design.

